# Supplementary material for: Carbon tax acceptability with information provision and mixed revenue uses
Source: Nat Commun. 2021 Dec 2;12:7017. doi: 10.1038/s41467-021-27380-8 (PMC8640071; doi:10.1038/s41467-021-27380-8)
Supplement: Supplementary file 1 — Supplementary Information [file 41467_2021_27380_MOESM1_ESM.pdf]

Supplementary Information

Supplementary Note 1. Context of carbon taxes in Spain

Spain has historically not had an explicit carbon tax on all products but has high levels of taxes on energy use. In 2014, Spain implicitly priced over 30% of the country's total carbon emissions above €30/ CO_2-ton, with the highest taxes generally being placed on the transportation sector[1]. In 2019, Spain again priced a similar percentage of around 30% of emissions at an average of €15/CO_2-ton, covering a mere 3% of its total emissions output[2]. Moreover, as a member of the European Union, Spain is covered by the EU ETS. According to the World Bank[3], Spain has a carbon tax since 2014 ($17 per ton of CO_2). However, this is a tax aimed at reducing fluorinated greenhouse gases (F-gases) (see the entry for Spain in the Carbon Pricing Dashboard of The World Bank: https://carbonpricingdashboard.worldbank.org/map_data). It can be interpreted as an implicit carbon price, but to call it an explicit carbon tax would be inaccurate. Moreover, there is hardly any discussion about carbon pricing in Spanish media or society. For example, an analysis of coverage of climate change mitigation in Spanish newspapers shows that although "economic incentives" receive some attention when discussing solutions, carbon taxation overall does not stand out as an important issue[4]. Note further that refereed reviews of carbon taxation tend not to mention Spain (e.g., Haites[5]).

Supplementary Note 2. Survey questions in English (translated from Spanish)

Under the Paris Agreement from 2015, each country, including Spain, must implement policies to reduce their CO_2 emissions, which contribute to climate change. One major proposal to achieve emissions reduction is by implementing a carbon tax on fossil fuels whose combustion is the main cause of CO_2 emissions.

1. Do you know how a carbon tax works? (Response options: not at all, a little, somewhat, a lot, very much)

2. Here are several sentences about carbon taxation. Can you tell us whether you think they are true or false? (Response options: "true", "false", "do not know")
   - A carbon tax is levied on the carbon content of fossil fuels, such as coal and oil.
   - A carbon tax mandates all producers and consumers which low-carbon technology they should adopt.
   - A carbon tax makes renewable energy sources, such as solar electricity, more expensive than fossil fuels.
   - A carbon tax imposes a legally binding limit on the amount of CO_2 emissions that firms and consumers are allowed to emit.

- A carbon tax allows reducing other, existing taxes such as VAT or labour taxes.
- A carbon tax will raise the price of coal and reduce the price of gasoline.

The following information is provided to half of the sample (group 1) about how a carbon tax works:

"A carbon tax is a charge on fossil fuels in proportion to the amount of carbon they contain as this determines how many CO_2 emissions result from their combustion. This will, for instance, raise the price of coal more than that of gasoline and the latter more than that of natural gas. Producers and consumers are then stimulated to switch to renewable energy, save energy on heating, alter fuel-based transport, etc. Because fuel prices alter, the prices of other products and services throughout the economy will change as well: the ones that generate considerable CO_2 in production will become more expensive, while prices are likely to alter little or remain the same for products and services that cause little or no CO_2 during production. A significant carbon tax thus encourages all firms and household to shift to goods and services that use fewer high-carbon energy sources during their production."

3. How effective do you think a carbon tax is for reducing CO_2 emissions? (Response options: Very ineffective, ineffective, neither ineffective nor effective, effective, very effective).
4. How fair or unfair do you consider a carbon tax? (Response options: very unfair, somewhat unfair, neither unfair nor fair, somewhat fair, very fair).
5. Who do you think should carry most of the burden of the carbon tax? (Response options: businesses, consumers, both, none).
6. How do you think a carbon tax will affect you personally? (Response options: I would be much worse off, I would be somewhat worse off, I would be neither worse off nor better off, I would be somewhat better off, I would be much better off).
7. How do you think a carbon tax will affect low-income households? (Response options: they would be much worse off, they would be somewhat worse off, they would be neither worse off nor better off, they would be somewhat better off, they would be much better off).
8. How trustworthy do you think politicians are in implementing the carbon tax properly? (Response options: very untrustworthy, untrustworthy, neither untrustworthy not trustworthy, trustworthy, very trustworthy).
9. How acceptable do you find a carbon tax? (Response options: completely unacceptable, somewhat unacceptable, neither unacceptable nor acceptable, somewhat acceptable, completely acceptable).
10. Which of these two objectives do you think is the main purpose of a carbon tax? (Response options: to generate revenues, to change behaviour of producers and consumers, do not know).

Carbon taxes generate revenues which can be used for different purposes. As an illustrative example, for instance, according to one estimate, a (low) carbon tax of €5/ton CO_2 would already generate approximately €1.3 billion of additional government revenues. To put this number in perspective: Spain´s expenditures for education were €2.6 billion in 2018.

Here we present five options to use the revenues:

- Return all the revenues to compensate low-income households.
- Support the development of climate projects (e.g. investing in public transport, planting trees, subsidies for renewable energy).
- Use half of the revenues to support the development of climate projects and the other half to compensate low-income households.
- Return the revenues in equal amount to all households as compensation.
- Use half of the revenues to support development of climate projects and the other half to compensate all households in equal amount.

11. How effective do you think the carbon tax is for reducing CO_2 emissions if its revenues are used to [the question is repeated for each of the five revenue uses mentioned above]? (Response options: Very ineffective, ineffective, neither ineffective nor effective, effective, very effective).

12. How fair or unfair do you consider a carbon tax if its revenues are used to [the question is repeated for each of the five revenue uses mentioned above]? (Response options: very unfair, somewhat unfair, neither unfair nor fair, somewhat fair, very fair).

13. How do you think a carbon tax affects you personally if its revenues are used to [the question is repeated for each of the five revenue uses mentioned above]? (Response options: I would be much worse off, I would be somewhat worse off, I would be neither worse off nor better off, I would be somewhat better off, I would be much better off).

14. How you think a carbon tax affects low-income households if its revenues are used to [the question is repeated for each of the five revenue uses mentioned above]? (Response options: they would be much worse off, they would be somewhat worse off, they would be neither worse off nor better off, they would be somewhat better off, they would be much better off).

15. How acceptable do you find the carbon tax if its revenues are used to [the question is repeated for each of the five revenue uses mentioned above]? (Response options: completely unacceptable, somewhat unacceptable, neither unacceptable nor acceptable, somewhat acceptable, completely acceptable).

16. What percentage of the total carbon tax revenues (100%) would you prefer to allocate for each of the 3 proposed options? Please make sure that the total amount is equal to 100%.

Revenue option	% of revenue allocated
Support the development of climate projects	
Return the revenues to compensate low-income households	
Return the revenues in equal amount to all households as compensation	[Computer does not allow them to proceed as long as three numbers do not add up to 100]

17. Which factor– effectiveness or fairness – played a stronger role in your decision on how to allocate the revenue generated by the carbon tax? (Response options: only effectiveness, more effectiveness than fairness, equally effectiveness and fairness, more fairness than effectiveness, only fairness, neither effectiveness nor fairness).
18. How many people are in your household?
19. Could you tell us in which of the following ranges your net monthly income of your household falls? (Responses options: No income, €1000 or less, between €1001-€2000, between €2001-€3000, between €3001-€4000, more than €4001, I prefer not to answer).
20. What is the highest level of studies you have completed? (Response options: less than 5 years of school, primary, secondary, medium professional formation, superior professional formation, university).
21. How concerned are you about climate change? (Response options: not at all, a little, somewhat, much, very much).
22. Where would you situate yourself ideologically? Use a scale ranging from 1 to 10, where 1 is 'left-wing' and 10 is 'right-wing'?
23. Which political party did you vote in the last general elections of the 28th April 2019?
24. How often do you use a car? (Response options: never, less than once a month, few times a month, once a week, few times a week, everyday).
25. How many minutes do you travel by car on an average working day? (Response options: none, less than 30 minutes, between 30 and 60 minutes, between 61 (1 hour) and 90 minutes (1:30 hours), Between 91 minutes (1:30hours) and 120 minutes (2 hours), more than 120 minutes (2hours or more).

Supplementary Note 3. Original survey questions in Spanish

Tal y como establece el Acuerdo de París del 2015, cada país, incluyendo España, debe implementar políticas para reducir sus emisiones de CO_2 que contribuyen al cambio climático. Una de las principales propuestas para conseguir esta reducción de emisiones es la implementación de un impuesto al carbono en los combustibles fósiles cuya combustión es la principal causa de emisiones de CO_2.

**1. ¿Sabes cómo funciona el impuesto al carbono?**	No	Un poco	Bastante	Mucho	Muchísimo

**2. A continuación, verás distintas frases sobre el impuesto al carbono ¿Piensas que las siguientes frases son ciertas o falsas?**	**Cierta**	**Falsa**	**No lo sé**
Un impuesto al carbono se aplica sobre el contenido de carbono de los combustibles fósiles, como el carbón y el petróleo			
Un impuesto al carbono establece qué tecnología baja en carbono deben adoptar todos los productores y consumidores			
Un impuesto al carbono hace que las fuentes de energía renovable, como la electricidad solar, sean más caras que los combustibles fósiles			
Un impuesto al carbono impone un límite legalmente vinculante a la cantidad de emisiones de CO_2 que las empresas y los consumidores pueden emitir			
Un impuesto al carbono permite reducir otros impuestos existentes, como el IVA o los impuestos laborales			
Un impuesto al carbono elevará el precio del carbón y reducirá el precio de la gasolina			

La siguiente información es sobre cómo funciona un impuesto al carbono:
Un impuesto al carbono es una tasa a los combustibles fósiles proporcional a la cantidad de carbono que contienen ya que esto determina las emisiones de CO_2 resultantes de su combustión. Esto aumentará, por ejemplo, el precio del carbón más que el de la gasolina y el de ésta más que el del gas natural. Como consecuencia, los productores y los

consumidores estarán incentivados a cambiar a energías renovables, ahorrar energía en calefacción y aire acondicionado, cambiar su medio de transporte basado en combustibles fósiles, etc. Debido a que los precios de los combustibles se alterarán, los precios de otros productos y servicios de casi toda la economía cambiaran también: los que generan una gran cantidad de CO_2 en la producción se volverán más caros, mientras que los precios de los productos y servicios que generan poco o nada de CO_2 durante su producción probablemente se modifiquen poco o sigan siendo los mismos. Por tanto, un impuesto al carbono significativo fomenta que todas las empresas y consumidores cambien a productos y servicios que usan menos fuentes de energía altas en contenido de carbono durante su producción.

**3. ¿Cómo de efectivo crees que sería un impuesto al carbono para reducir las emisiones de CO_2?**	Muy inefectivo	Inefectivo	Ni inefectivo ni efectivo	Efectivo	Muy efectivo

**4. ¿Qué tan justo o injusto consideras que es un impuesto al carbono?**	Muy injusto	Algo injusto	Ni injusto ni justo	Algo justo	Muy justo

**5. ¿Quién piensas que debería asumir la mayor parte de la carga del impuesto al carbono?**	Empresas	Consumidores	Los dos	Ninguno

**6. ¿Cómo crees que te afectaría un impuesto al carbono?**	Me perjudicaría mucho	Me perjudicaría un poco	Ni me perjudicaría ni me beneficiaría	Me beneficiaría un poco	Me beneficiaría mucho

**7. ¿Cómo crees que un impuesto al carbono afectaría a los hogares de bajos ingresos?**	Les perjudicaría mucho	Les perjudicaría un poco	Ni les perjudicaría ni les beneficiaría	Les beneficiaría un poco	Les beneficiaría mucho

**8. ¿Qué tan fiables crees que serían los políticos para implementar el impuesto al carbono adecuadamente?**	Nada fiables	Poco fiables	Algo fiables	Fiables	Muy fiables

**9. ¿Cómo de aceptable encuentras un impuesto al carbono?**	Completamente inaceptable	Algo inacceptable	Ni inaceptable ni aceptable	Algo aceptable	Muy aceptable

**10. ¿Cuál de estos dos objetivos crees que es el principal de un impuesto al carbono?**	Generar ingresos	Cambiar el comportamiento de los productores y los consumidores	No lo sé

Los impuestos al carbono generan ingresos que pueden utilizarse para diferentes propósitos. A modo de ilustración, por ejemplo, según una estimación, un impuesto (bajo) al carbono de 5€/tonelada de CO_2 generaría aproximadamente 1.300 millones de euros de ingresos adicionales del gobierno. Para poner este número en perspectiva: los gastos de educación en España fueron de 2.600 millones de euros en 2018.

A continuación, verás 5 opciones para utilizar los ingresos:
a) Devolver todos los ingresos a los hogares de bajos ingresos como compensación.
b) Apoyar el desarrollo de proyectos climáticos (por ejemplo, invertir en transporte público, plantar árboles, subsidios para energías renovables).
c) Usar la mitad de los ingresos para apoyar el desarrollo de proyectos climáticos y la otra mitad para compensar a los hogares de bajos ingresos.
d) Devolver los ingresos en igual cantidad a todos los hogares como compensación.
e) Usar la mitad de los ingresos para apoyar el desarrollo de proyectos climáticos y la otra mitad para compensar a todos los hogares en igual cantidad.

Responda las siguientes preguntas sobre el impuesto al carbono dependiendo de cada uno de los cinco usos de los ingresos mencionados anteriormente:

**11. ¿Cómo de efectivo crees que sería el impuesto al carbono para reducir las emisiones de CO_2 si los ingresos del impuesto se utilizaran para...?**					
**Opción de uso de los ingresos**					
a) Devolver todos los ingresos a los hogares de bajos ingresos como compensación	Muy inefectivo	Inefectivo	Ni inefectivo ni efectivo	Efectivo	Muy efectivo
b) Apoyar el desarrollo de proyectos climáticos	Muy inefectivo	Inefectivo	Ni inefectivo ni efectivo	Efectivo	Muy efectivo
c) Usar la mitad de los ingresos para apoyar el desarrollo de proyectos climáticos y la otra mitad para compensar a los hogares de bajos ingresos	Muy inefectivo	Inefectivo	Ni inefectivo ni efectivo	Efectivo	Muy efectivo
d) Devolver los ingresos en igual cantidad a todos los hogares como compensación	Muy inefectivo	Inefectivo	Ni inefectivo ni efectivo	Efectivo	Muy efectivo
e) Usar la mitad de los ingresos para apoyar el desarrollo de proyectos climáticos y la otra mitad para	Muy inefectivo	Inefectivo	Ni inefectivo ni efectivo	Efectivo	Muy efectivo

compensar a todos los hogares en igual cantidad					

**12. ¿Qué tan justo o injusto consideras un impuesto al carbono si los ingresos del impuesto se utilizaran para...?**					
**Opción de uso de los ingresos**					
a) Devolver todos los ingresos a los hogares de bajos ingresos como compensación	Muy injusto	Injusto	Ni injusto ni justo	Justo	Muy justo
b) Apoyar el desarrollo de proyectos climáticos	Muy injusto	Injusto	Ni injusto ni justo	Justo	Muy justo
c) Usar la mitad de los ingresos para apoyar el desarrollo de proyectos climáticos y la otra mitad para compensar a los hogares de bajos ingresos	Muy injusto	Injusto	Ni injusto ni justo	Justo	Muy justo
d) Devolver los ingresos en igual cantidad a todos los hogares como compensación	Muy injusto	Injusto	Ni injusto ni justo	Justo	Muy justo
e) Usar la mitad de los ingresos para apoyar el desarrollo de proyectos climáticos y la otra mitad para compensar a todos los hogares en igual cantidad	Muy injusto	Injusto	Ni injusto ni justo	Justo	Muy justo

**13. ¿Cómo crees que te afectaría un impuesto al carbono si los ingresos del impuesto se utilizaran para...?**					
**Opción de uso de los ingresos**					
a) Devolver todos los ingresos a los hogares de bajos ingresos como compensación	Me perjudicaría mucho	Me perjudicaría un poco	Ni me perjudicaría ni me beneficiaría	Me beneficiaría un poco	Me beneficiaría mucho
b) Apoyar el desarrollo de proyectos climáticos	Me perjudicaría mucho	Me perjudicaría un poco	Ni me perjudicaría ni me beneficiaría	Me beneficiaría un poco	Me beneficiaría mucho
c) Usar la mitad de los ingresos para apoyar el desarrollo de proyectos climáticos y la otra mitad para compensar a los hogares de bajos ingresos	Me perjudicaría mucho	Me perjudicaría un poco	Ni me perjudicaría ni me beneficiaría	Me beneficiaría un poco	Me beneficiaría mucho
d) Devolver los ingresos en	Me	Me	Ni me	Me	Me

igual cantidad a todos los hogares como compensación	perjudicaría mucho	perjudicaría un poco	perjudicaría ni me beneficiaría	beneficiaría un poco	beneficiaría mucho
e) Usar la mitad de los ingresos para apoyar el desarrollo de proyectos climáticos y la otra mitad para compensar a todos los hogares en igual cantidad	Me perjudicaría mucho	Me perjudicaría un poco	Ni me perjudicaría ni me beneficiaría	Me beneficiaría un poco	Me beneficiaría mucho

**14. ¿Cómo crees que un impuesto al carbono afectaría a las personas de bajos ingresos si los ingresos del impuesto se utilizaran para...?**					
**Opción de uso de los ingresos**					
a) Devolver todos los ingresos a los hogares de bajos ingresos como compensación	Les perjudicaría mucho	Les perjudicaría un poco	Ni les perjudicaría ni les beneficiaría	Les beneficiaría un poco	Les beneficiaría mucho
b) Apoyar el desarrollo de proyectos climáticos	Les perjudicaría mucho	Les perjudicaría un poco	Ni les perjudicaría ni les beneficiaría	Les beneficiaría un poco	Les beneficiaría mucho
c) Usar la mitad de los ingresos para apoyar el desarrollo de proyectos climáticos y la otra mitad para compensar a los hogares de bajos ingresos	Les perjudicaría mucho	Les perjudicaría un poco	Ni les perjudicaría ni les beneficiaría	Les beneficiaría un poco	Les beneficiaría mucho
d) Devolver los ingresos en igual cantidad a todos los hogares como compensación	Les perjudicaría mucho	Les perjudicaría un poco	Ni les perjudicaría ni les beneficiaría	Les beneficiaría un poco	Les beneficiaría mucho
e) Usar la mitad de los ingresos para apoyar el desarrollo de proyectos climáticos y la otra mitad para compensar a todos los hogares en igual cantidad	Les perjudicaría mucho	Les perjudicaría un poco	Ni les perjudicaría ni les beneficiaría	Les beneficiaría un poco	Les beneficiaría mucho

**15. ¿Cómo de aceptable encuentras el impuesto al carbono si los ingresos del impuesto se utilizaran para...?**					
**Opción de uso de los ingresos**					
a) Devolver todos los ingresos a los hogares de bajos ingresos como compensación	Completamente inaceptable	Inaceptable	Ni inaceptable ni aceptable	Aceptable	Completamente aceptable

b) Apoyar el desarrollo de proyectos climáticos	Completamente inaceptable	Inaceptable	Ni inaceptable ni aceptable	Aceptable	Completamente aceptable
c) Usar la mitad de los ingresos para apoyar el desarrollo de proyectos climáticos y la otra mitad para compensar a los hogares de bajos ingresos	Completamente inaceptable	Inaceptable	Ni inaceptable ni aceptable	Aceptable	Completamente aceptable
d) Devolver los ingresos en igual cantidad a todos los hogares como compensación	Completamente inaceptable	Inaceptable	Ni inaceptable ni aceptable	Aceptable	Completamente aceptable
e) Usar la mitad de los ingresos para apoyar el desarrollo de proyectos climáticos y la otra mitad para compensar a todos los hogares en igual cantidad	Completamente inaceptable	Inaceptable	Ni inaceptable ni aceptable	Aceptable	Completamente aceptable

16. ¿Qué porcentaje del total de los ingresos del impuesto al carbono (100%) preferirías que se usara para cada una de las 3 opciones propuestas? Por favor, asegúrate de que la suma total sea igual a 100%.

**Opción de uso de los ingresos**	**% de los ingresos asignados**
Apoyar el desarrollo de proyectos climáticos	
Devolver los ingresos a los hogares de bajos ingresos como compensación	
Devolver los ingresos en igual cantidad a todos los hogares como compensación	

17. ¿Qué factor, la efectividad o la justicia, ha desempeñado un papel más importante en tu decisión sobre cómo usar los ingresos generados por el impuesto al carbono?

**Solo la efectividad**	**Más la efectividad que la justicia**	**Igual importancia la efectividad y la justicia**	**Más la justicia que la efectividad**	**Solo la justicia**	**Ni la efectividad ni la justicia**

**18. ¿Cuántas personas viven en tu hogar?**	

**19. ¿En cuál de los siguientes rangos está el ingreso neto mensual de tu hogar?**						
Sin ingresos	1000€ o menos	Entre 1001€ - 2000€	Entre 2001€ - 3000€	Entre 3001€ - 4000€	Más de 4000€	Prefiero no contestar

**20. ¿Cuáles son los estudios de más alto nivel que has completado?**					
Menos de 5 años de escolarización	Educación primaria	Educación secundaria	Bachillerato/Formación Profesional (FP) de grado medio	FP de grado superior	Estudios universitarios/Estudios de postgrado y doctores

**21. ¿Estás preocupado/a por el cambio climático?**	No	Un poco	Bastante	Mucho	Muchísimo

**22. ¿Dónde te situarías ideológicamente? Utiliza una escala del 1 al 10, siendo 1 "Izquierda" y 10 "Derecha"**	1 Izquierda	2	3	4	5	6	7	8	9	10 Derecha	No sé/ Prefiero no contestar

**23. ¿Podrías indicar a qué partido político votaste en las anteriores elecciones generales del 28 de abril de 2019?**									
	1 PSOE	2 PP	3 Ciudadanos	4 Unidas Podemos	5 Vox	6 ERC-Sobiranistes		7 JxCat	8 EAJ-PNV
	9 EH Bildu	10 CC-PNC	11 NA+	12 Compromís	13 PRC	14 "Otros" Especificar	15 Voté en blanco	16 Voté nulo	17 No voté en estas elecciones

**24. ¿Con qué frecuencia usas el coche?**	Nunca	Menos de una vez al mes	Unas pocas veces al mes	Una vez a la semana	Unas pocas veces a la semana	Cada día
**25. ¿Cuántos minutos de promedio viajas en automóvil en un día laboral?**	Nada	Menos de 30 minutos	Entre 30 y 60 minutos	Entre 61 minutos (1 hora) y 90 minutos (1 hora y media)	Entre 91 minutos (1 hora y media) y 120 minutos (2 horas)	Más de 120 minutos (2 horas o más)

Supplementary Table 1. Descriptive statistics on sociodemographic variables.

Variables	Description	Mean (SD) or %	Spanish population
Gender	Dummy: female	51.1%	51.1%
Age	18 to 87 years old	45.15 (15.18)	44.2
Household size	Number of people living in respondent's house	2.96 (1.21)	2.5
Monthly household income	1 (No income) to 6 (More than 4000€)	3.73 (1.12) translating into a mean between €2200 and 2700	2295 euros per month
Education	1 (Less than 5 years of school) to 6 (University)	5.02 (1.16); 89.92% of the sample have medium professional or higher studies	60.2% have a medium professional or higher studies (CIS, 2019)*
Political orientation	1 (left-wing) to 10 (right-wing)	4.47 (2.45)	4.5 (2.1) (CIS, 2019)*

Notes: Sampling was done by using quotas on age, gender and geographical distribution, making the survey sample representative of the general population on these characteristics. The rest of variables are compared with census data from the Spanish National Institute of Statistics (www.ine.es) unless other source is indicated. The results show that the sample is also representative for the other covariates, except for the variable education. Our sample has higher education than the average of the Spanish population. The sample includes N=2004 respondents for all variables except for monthly household income (N=1572) and political orientation (N=1741) because of missing observations.

* Source: Centro de Investigaciones Sociológicas (CIS), Barómetro de Septiembre 2019.

Supplementary Table 2. Descriptive statistics on covariates for treatment and control groups

	**Mean in experiment group**	**Mean in control group**	**Kruskal-Wallis rank sum test p-value**
Climate concern	3.50	3.53	0.52
Education	4.98	5.06	0.14
Monthly household income	3.74	3.72	0.86
Political orientation	4.47	4.47	0.95
Trust in politicians	1.67	1.62	0.11
Self-perceived knowledge	1.56	1.52	0.51
Assessed knowledge	2.11	2.18	0.19
Household size	3.00	2.91	0.12
Car use	4.47	4.52	0.29

Notes: We use quotas on age, gender and geographical distribution to ensure that treatment and control groups have a similar distribution regarding these three covariates. We compared the two subsamples using the Kruskal-Wallis rank sum test with Bonferroni correction, taking into account other covariates, and showing that these are not statistically different (p-values always above 0.1). The sample includes N=2004 respondents split among the experimental group (n=1004 respondents) and the control group (n=1000 respondents) for all covariates except for political orientation (n=803 in the experimental group and N=769 in the control group) and monthly household income (n=871 in the experimental group and N=870 in the control group).

Supplementary Table 3. Interaction between assessed knowledge and information provision for unspecified revenue use based on ordered logit regression.

Explanatory variables	Acceptability
Self-perceived knowledge	0.96<br>(0.86-1.08)
Assessed knowledge*Information	1.10***<br>(1.05-1.15)
Age	1.00<br>(0.99-1.00)
Gender	0.96<br>(0.81-1.14)
Education	1.06<br>(0.98-1.14)
Climate concern	1.59***<br>(1.48-1.72)
Political orientation	-0.90***<br>(0.88-0.92)
Monthly income	1.00<br>(0.95-1.05)
Car use	0.88***<br>(0.84-0.92)
Trust in politicians	2.07***<br>(1.87-2.31)
Household size	0.88***<br>(0.82-0.95)
Nagelkerke pseudo R^2	0.75

Note: Coefficients indicate odds ratios with 2.5%-97.5% confidence intervals expressed within brackets. Asterisks ***, **, and * indicate 1%, 5%, and 10% significance, respectively.

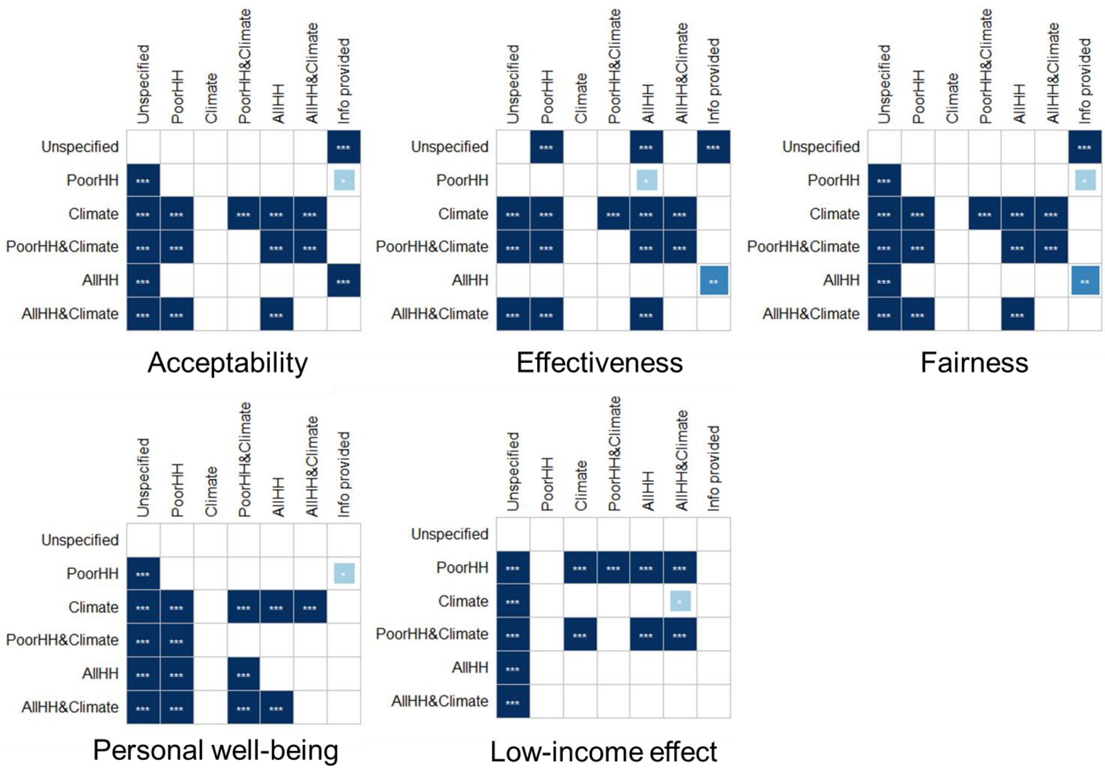

Supplementary Figure 1. Tests for stochastic dominance in respondents' perception under different carbon tax revenue use and information provision. The first six columns of the matrices represent the results of the pairwise Mann-Whitney test. The dark shading indicates that perception of respondent on a given characteristic from the revenue use specified in the respective row dominates the perception of the revenue use in the corresponding column with a given significance level. The last column summarizes results of the Kruskal-Wallis rank sum test with Bonferroni correction. Again, the dark shading indicates that perceptions with information provision on carbon tax are significantly larger than those without the information being provided. Asterisks ***, **, and * denote 1%, 5%, and 10% significance, respectively. The sample includes N=2004 respondents for each revenue use option. When comparing the sub-samples with and without the information provided (variable Info provided), the former sub-sample has n=1004 informed respondents and the latter has n=1000 non-informed respondents.

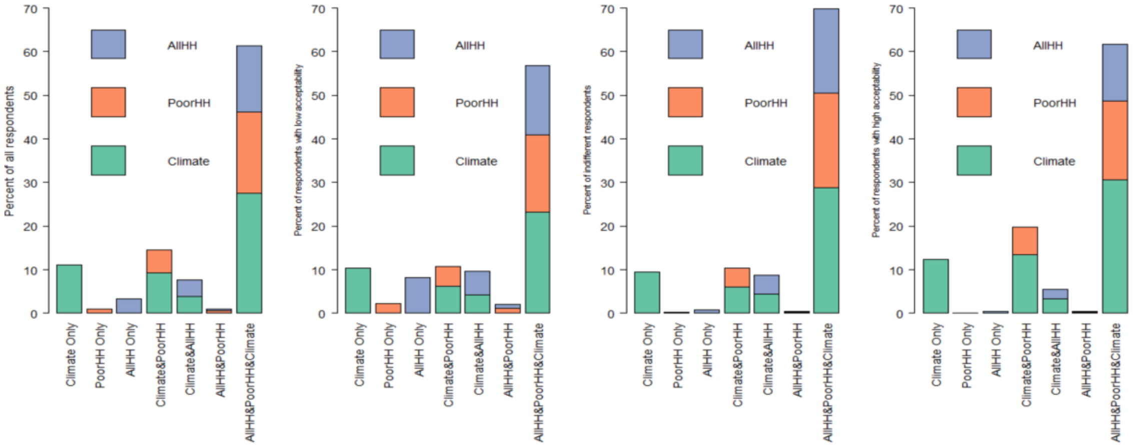

Supplementary Figure 2. Preferred allocation of revenues from a carbon tax. Respondents were asked to express the share (in %) of total revenues to be allocated to each revenue use. The left chart is for all respondents, the central-left chart is for respondents who initially did not accept a carbon tax, the central-right chart is for initially indifferent respondents and the right chart is for respondents who initially accepted a carbon tax. Respondents with high (low) acceptability are defined as having a response of higher (lower) than 3 (on a scale from 1 to 5) and indifferent respondents are those that chose the median (3) option. All respondents expressed their acceptability before any revenue use was specified. The coloured parts of each bar indicate average shares. Note that our overall sample size is 2004 (first chart), number of respondents who did not accept a carbon tax is 756, number of respondents who are indifferent is 387, and number of respondents who accepted a carbon tax is 861.

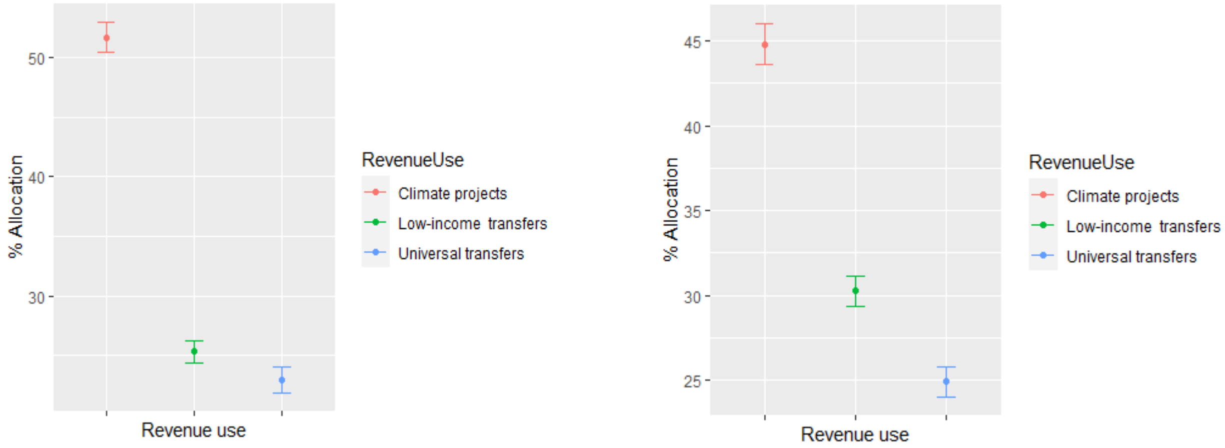

Supplementary Figure 3. Allocation of revenues between different options. Dots indicate the average results with error bars indicating +/- 2 s.e. The left chart is for all 2004 respondents, while the right one is for those who allocated non-zero values for all three options (N=1231).

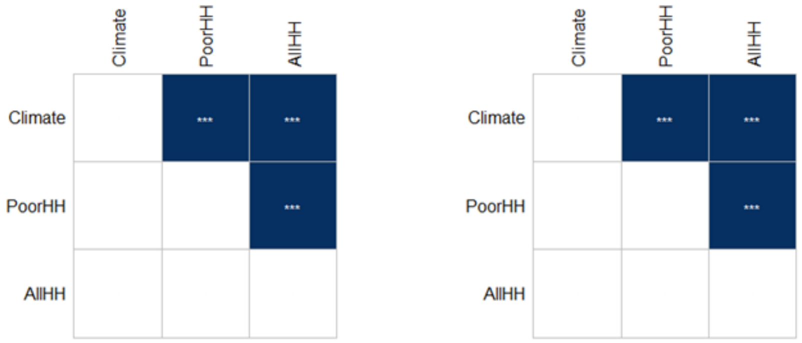

Supplementary Figure 4. Tests for stochastic dominance in respondents' allocation of revenues between different options. The columns of the matrices represent the results of the pairwise one-sided Mann-Whitney test. Dark shading indicates that revenue allocation for an option dominates the allocation of revenues in the corresponding column with a given significance level. Asterisk *** indicate 1% significance. N=2004 respondents.

Supplementary Note 4. Interpretation of coefficient estimates for sociodemographic variables in Table 1

Regarding sociodemographic variables, our results show that individuals with a higher self-perceived knowledge have a lower probability to accept a carbon tax and consider it as less effective and more unfair. In addition, younger and female respondents, people with higher climate concern, with more left-wing political orientation, using their car less often and with more trust in politicians tend to exhibit significantly higher acceptability of the carbon tax policy. Other controls, such as monthly income and the size of a household, do not have a significant effect on acceptability. Similar results are found for the influence of these variables on perceived effectiveness and fairness.

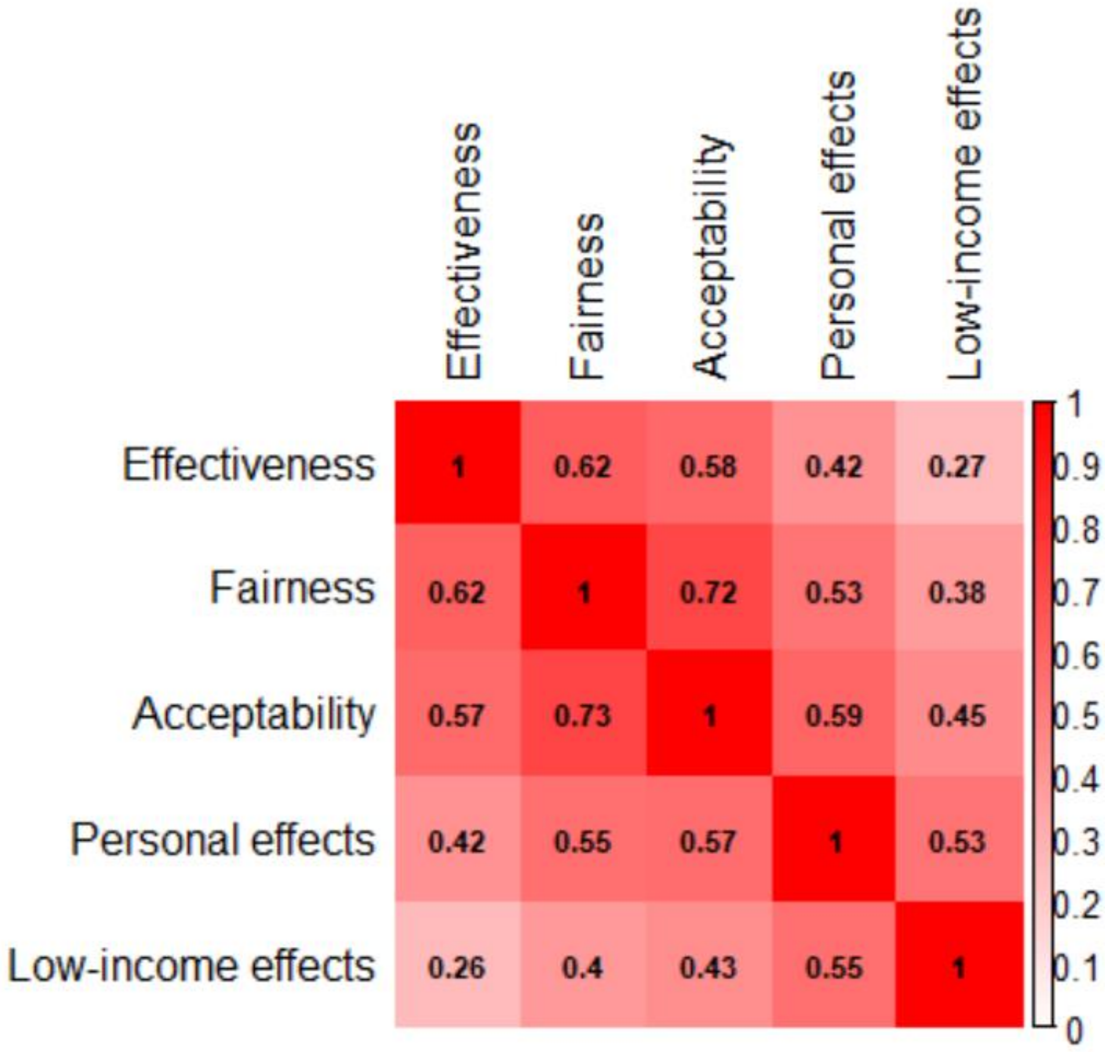

Supplementary Figure 5. Matrix of correlations between the perception indicators of carbon taxation. Entries below the diagonal are Pearson correlation coefficients, and above the diagonal are Spearman rank correlation coefficients. N= 2004 respondents.

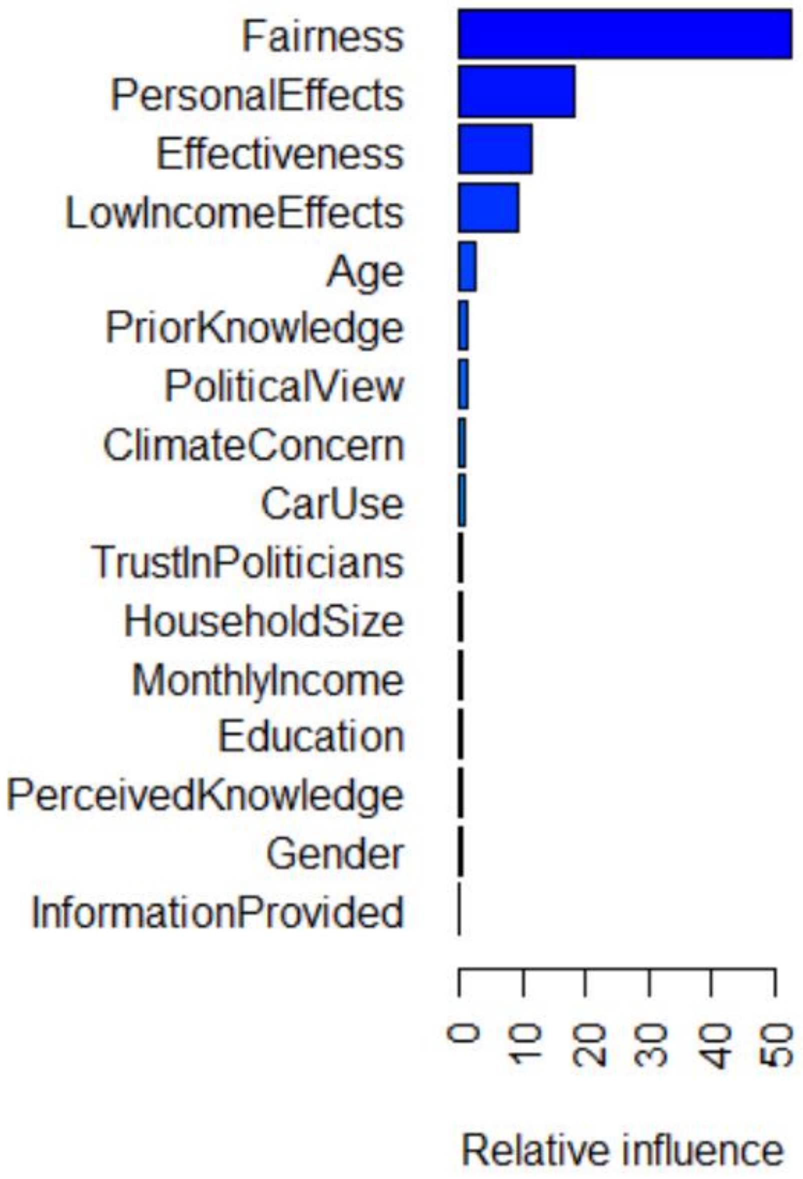

Supplementary Figure 6. Results from applying GBM on predictors of acceptability listed in Table 2. The variables are ranked in their relative influence (in %) in 1000 regression trees.

Supplementary Table 4. Determinants of acceptability under different revenue uses based on ordered logit regression.

**Explanatory variables**	**Unspecified revenue use**	**PoorHH**	**Climate**	**PoorHH& Climate**	**AllHH**	**AllHH& Climate**
Self-perceived knowledge	0.96 (0.83-1.10)	0.87* (0.76-1.00)	0.83** (0.72-0.97)	0.83** (0.72-0.96)	0.83*** (0.72-0.95)	0.81*** (0.70-0.93)
Assessed knowledge	1.09** (1.01-1.18)	0.93* (0.86-1.01)	1.26*** (1.16-1.37)	1.14 (1.05-1.23)	0.93* (0.86-1.01)	1.08* (1.00-1.17)
Information provision	1.34*** (1.11-1.62)	1.09 (0.91-1.32)	1.05 (0.86-1.28)	1.14 (0.94-1.38)	1.21** (1.00-1.47)	1.05 (0.87-1.27)
Age	0.99 (0.99-1.00)	0.99 (0.99-1.00)	1.01** (1.00-1.01)	0.99 (0.98-1.00)	0.99** (0.98-1.00)	1.00 (0.99-1.00)
Gender	0.87 (0.71-1.07)	1.09 (0.88-1.33)	1.02 (0.83-1.26)	1.09 (0.89-1.35)	1.39*** (1.13-1.71)	1.48*** (1.20-1.82)
Education	1.05 (0.96-1.15)	0.95 (0.87-1.04)	1.00 (0.92-1.10)	0.94 (0.86-1.03)	0.92* (0.84-1.01)	0.93 (0.85-1.02=
Climate concern	1.64*** (1.49-1.79)	1.07*** (0.98-1.17)	1.87*** (1.70-2.06)	1.42*** (1.30-1.56)	0.94 (0.86-1.02)	1.20*** (1.10-1.31)
Political orientation	0.87*** (0.84-0.91)	0.86*** (0.82-0.89)	0.88*** (0.84-0.92)	0.87*** (0.83-0.90)	0.99 (0.96-1.04)	0.98 (0.94-1.02)
Monthly income	1.18*** (1.08-1.3)	0.86** (0.78-0.94)	1.08 (0.98-1.19)	0.88** (0.80-0.97)	0.93 (0.84-1.02)	0.97 (0.88-1.07)
Car use	0.85*** (0.80-0.90)	0.99 (0.93-1.05)	0.89*** (0.84-0.95)	0.92*** (0.86-0.97)	0.99 (0.93-1.05)	0.94* (0.89-1.00)
Trust in politicians	2.08*** (1.84-2.36)	1.13** (1.01-1.28)	1.22*** (1.07-1.38)	1.25*** (1.10-1.41)	1.10 (0.97-1.24)	1.20*** (1.06-1.36)
Household size	0.87*** (0.80-0.95)	1.15** (1.06-1.24)	0.93 (0.86-1.01)	1.05 (0.97-1.14)	1.11** (1.02-1.21)	1.05 (0.97-1.14)
Nagelkerke pseudo R^2	0.81	0.74	0.77	0.74	0.73	0.72

Note: Coefficients indicate odds ratios with 2.5%-97.5% confidence intervals expressed within brackets. Asterisks ***, **, and * indicate 1%, 5%, and 10% significance, respectively.

Supplementary Table 5. Determinants of acceptability and perceptions. Robustness test when controls on monthly income and political orientation are dropped. Based on ordered logit regression.

**Explanatory variables**	**Acceptability**	**Perceptions**			
		**Effectiveness**	**Fairness**	**Low-income effects**	**Personal effects**
**Revenue uses**					
PoorHH	3.38*** (2.48-4.61)	1.32* (0.98-1.79)	2.25*** (1.66-3.06)	28.25*** (20.29-39.39)	4.8*** (3.53-6.54)
Climate	3.73*** (2.73-5.10)	1.94*** (1.43-2.62)	2.94*** (2.16-4.01)	14.72*** (10.68-20.34)	8.03*** (5.87-11.01)
PoorHH&Climate	2.90*** (2.13-3.94)	1.38** (1.03-1.87)	2.41*** (1.78-3.27)	20.77*** (15.07-28.68)	5.25*** (3.86-7.15)
AllHH	3.44*** (2.52-4.68)	1.55*** (1.14-2.09)	2.37*** (1.74-3.22)	15.89*** (11.52-21.93)	7.87*** (5.77-10.75)
AllHH&Climate	3.15*** (2.32-4.29)	1.42** (1.05-1.91)	2.53*** (1.86-3.44)	13.85*** (10.08-19.07)	7.53*** (5.52-10.28)
**Interactions between Assessed knowledge and Revenue uses**					
Assessed knowledge*Unspecified	1.19*** (1.12-1.26)	1.02 (0.97-1.08)	1.02 (0.96-1.08)	0.99 (0.93-1.06)	0.97 (0.91-1.03)
Assessed knowledge*PoorHH	0.92** (0.87-0.98)	0.87*** (0.82-0.93)	0.90*** 0.85-0.96)	1.27*** (1.19-1.35)	0.89*** (0.84-0.94)
Assessed knowledge*Climate	1.32*** (1.24-1.40)	1.23*** (1.16-1.31)	1.29*** (1.21-1.37)	1.11*** (1.05-1.18)	1.27*** (1.19-1.35)
Assessed knowledge*PoorHH&Climate	1.13*** (1.06-1.19)	1.11*** (1.05-1.18)	1.08** (1.02-1.15)	1.15*** (1.09-1.22)	1.07** (1.01-1.14)
Assessed knowledge*AllHH	0.91*** (0.86-0.97)	0.81*** (0.76-0.86)	0.87*** (0.82-0.93)	1.03 (0.97-1.10)	1.02 (0.96-1.08)
Assessed knowledge*AllHH&Climate	1.06 (0.99-1.12)	1.03 (0.97-1.10)	1.00 (0.94-1.06)	1.08 (1.02-1.14)	1.10*** (1.04-1.17)
**Interactions between Information provision and Revenue uses**					
Information*Unspecified	1.34*** (1.14-1.58)	1.34*** (1.15-1.56)	1.41*** (1.19-1.66)	0.98 (0.82-1.15)	0.92 (0.78-1.08)
Information*PoorHH	1.14 (0.97-1.33)	1.11 (0.95-1.30)	1.13 (0.97-1.32)	0.93 (0.78-1.10)	1.12 (0.96-1.31)
Information*Climate	1.01 (0.86-1.19)	0.96 (0.82-1.13)	0.97 (0.83-1.14)	0.92 (0.78-1.07)	1.02 (0.86-1.20)
Information*PoorHH&Climate	1.13 (0.97-1.32)	1.08 (0.92-1.26)	0.99 (0.85-1.16)	0.93 (0.80-1.09)	1.06 (0.90-1.24)
Information*AllHH	1.21** (1.03-1.42)	1.22** (1.03-1.43)	1.18** (1.01-1.38)	1.10 (0.94-1.29)	1.04 (0.88-1.22)
Information*AllHH&Climate	1.08 (0.92-1.26)	1.02 (0.87-1.19)	1.00 (0.86-1.17)	1.07 (0.92-1.25)	0.97 (0.82-1.14)
**Control variables**					
Self-perceived knowledge	0.87*** (0.83-0.92)	0.90*** (0.86-0.95)	0.84*** (0.80-0.88)	0.89*** (0.84-0.93)	0.89*** (0.85-0.93)
Age	0.99*** (0.99-1.00)	0.99*** (0.99-1.00)	0.99*** (0.99-1.00)	1.00** (1.00-1.01)	1.00** (0.99-1.00)
Gender	1.16*** (1.08-1.24)	1.20*** (1.12-1.28)	1.12*** (1.04-1.20)	1.16*** (1.08-1.24)	1.12*** (1.04-1.20)
Education	0.93*** (0.91-0.96)	0.92*** (0.89-0.95)	0.92*** (0.90-0.95)	0.95*** (0.92-0.98)	0.94*** (0.91-0.97)
Climate concern	1.38*** (1.34-1.42)	1.22*** (1.19-1.26)	1.31*** (1.27-1.35)	1.33*** (1.29-1.37)	1.36*** (1.32-1.40)
Car use	0.93*** (0.91-0.95)	0.97*** (0.95-0.99)	0.95*** (0.93-0.97)	0.95*** (0.93-0.97)	0.92*** (0.90-0.94)
Trust in politicians	1.34*** (1.28-1.39)	1.33*** (1.28-1.39)	1.24*** (1.19-1.29)	1.26*** (1.21-1.32)	1.31*** (1.26-1.37)
Household size	0.98 (0.95-1.01)	1.00 (0.97-1.03)	0.96** (0.94-0.99)	0.98 (0.95-1.01)	0.95 (0.93-0.98)
Nagelkerke pseudo R^2	0.72	0.72	0.73	0.79	0.74

Note: Coefficients indicate odds ratios with 2.5%-97.5% confidence intervals expressed within brackets. Asterisks ***, **, and * indicate 1%, 5%, and 10% significance, respectively.

Supplementary Table 6. Determinants of fairness and acceptability. Robustness test when controls on monthly income and political orientation are dropped. Based on ordered logit regression.

Explanatory variables	Perceived fairness	Acceptability	
**Perceptions**			
Effectiveness	-	2.57^{***} (2.47-2.67)	1.66^{***} (1.60-1.73)
Fairness	-	-	4.81^{***} (4.59-5.05)
Personal effects	2.82^{***} (2.71-2.94)	2.37^{***} (2.27-2.47)	-
Low-income effects	1.24^{***} (1.20-1.28)	1.35^{***} (1.31-1.40)	-
**Control variables**			
Self-perceived knowledge	0.88^{***} (0.83-0.92)	0.94^{**} (0.89-0.99)	0.98^{**} (0.92-1.03)
Assessed knowledge	0.99 (0.97-1.02)	1.07^{***} (1.04-1.10)	1.10^{***} (1.07-1.13)
Information provided	1.10^{***} (1.03-1.18)	1.14^{***} (1.06-1.22)	1.11^{**} (1.03-1.19)
Age	0.99^{***} (0.99-1.00)	1.00^{*} (0.99-1.00)	1.00 (0.99-1.00)
Gender	1.08^{*} (1.01-1.16)	1.03 (0.96-1.11)	1.03 (0.95-1.11)
Education	0.94^{***} (0.91-0.97)	0.99 (0.96-1.02)	1.00 (0.97-1.03)
Climate concern	1.11^{***} (1.07-1.14)	1.17^{***} (1.13-1.21)	1.21^{***} (1.17-1.25)
Car use	0.99 (0.97-1.01)	0.96 (0.94-0.98)	0.95^{***} (0.93-0.97)
Trust in politicians	1.06^{**} (1.02-1.11)	1.07^{***} (1.02-1.12)	1.14^{***} (1.09-1.19)
Household size	0.99 (0.96-1.02)	1.00 (0.97-1.03)	1.00 (0.97-1.03)
Nagelkerke pseudo R^2	0.78	0.84	0.85

Note: Coefficients indicate odds ratios with 2.5%-97.5% confidence intervals expressed within brackets. Asterisks ***, **, and * denote 1%, 5%, and 10% significance, respectively.

References

1. OECD. Effective Carbon Rates 2018: pricing carbon emissions through taxes and emissions trading. *OECD Publishing* (2018).

2. Pettigrew S. Spanish carbon pricing the lowest in the EU. https://www.climatescorecard.org/2020/03/spanish-carbon-pricing-the-lowest-in-the-eu/ (2020).

3. World Bank. "State and Trends of Carbon Pricing – 2019." Washington, DC (2019).

4. Fernández-Reyes, R., and Jiménez Gómez, I. Media coverage of climate change mitigation in the Spanish press. In: La comunicación de la mitigación ante la emergencia climática. ISBN 9788418167034, p. 207-246 (2019).

5. Haites, E. Carbon taxes and greenhouse gas emissions trading systems: what have we learned? Climate Policy 18(8): 955-966 (2018).
