# Supplementary material for: Carbon tax acceptability with information provision and mixed revenue uses
Source: Nat Commun. 2021 Dec 2;12:7017. doi: 10.1038/s41467-021-27380-8 (PMC8640071; doi:10.1038/s41467-021-27380-8)
Supplement: Supplementary file 3 — Description of Additional Supplementary Files [file 41467_2021_27380_MOESM3_ESM.pdf]

Description of Additional Supplementary Files

File Name: Supplementary Software 1

Description: R codes
