# Supplementary material for: Carbon tax acceptability with information provision and mixed revenue uses
Source: Nat Commun. 2021 Dec 2;12:7017. doi: 10.1038/s41467-021-27380-8 (PMC8640071; doi:10.1038/s41467-021-27380-8)
Supplement: Supplementary file 5 — Reporting Summary [file 41467_2021_27380_MOESM5_ESM.pdf]

nature research

Corresponding author(s): Sara Maestre Andrés

Last updated by author(s): Oct 8, 2021

Reporting Summary

Nature Research wishes to improve the reproducibility of the work that we publish. This form provides structure for consistency and transparency in reporting. For further information on Nature Research policies, see our Editorial Policies and the Editorial Policy Checklist.

Statistics

For all statistical analyses, confirm that the following items are present in the figure legend, table legend, main text, or Methods section.

n/a	Confirmed	
☐	☒	The exact sample size (*n*) for each experimental group/condition, given as a discrete number and unit of measurement
☐	☒	A statement on whether measurements were taken from distinct samples or whether the same sample was measured repeatedly
☐	☒	The statistical test(s) used AND whether they are one- or two-sided<br>*Only common tests should be described solely by name; describe more complex techniques in the Methods section.*
☐	☒	A description of all covariates tested
☐	☒	A description of any assumptions or corrections, such as tests of normality and adjustment for multiple comparisons
☐	☒	A full description of the statistical parameters including central tendency (e.g. means) or other basic estimates (e.g. regression coefficient) AND variation (e.g. standard deviation) or associated estimates of uncertainty (e.g. confidence intervals)
☐	☒	For null hypothesis testing, the test statistic (e.g. *F*, *t*, *r*) with confidence intervals, effect sizes, degrees of freedom and *P* value noted<br>*Give P values as exact values whenever suitable.*
☒	☐	For Bayesian analysis, information on the choice of priors and Markov chain Monte Carlo settings
☒	☐	For hierarchical and complex designs, identification of the appropriate level for tests and full reporting of outcomes
☐	☒	Estimates of effect sizes (e.g. Cohen's *d*, Pearson's *r*), indicating how they were calculated

Our web collection on statistics for biologists contains articles on many of the points above.

Software and code

Policy information about availability of computer code

Data collection	Data collection has been conducted by a professional survey company "Netquest".
Data analysis	Data analysis has been done in R software (version 4.0.3). The code is available in the supplementary material. To rank predictors of acceptability in terms of predictive power, we use a gradient boosting machines (GBM, version 2.1.8) algorithm implemented in the software R. To validate the model, we use a 10-fold cross-validation. This means that GBM splits the data ten times randomly into training and testing sets, trains the model on the training data and then evaluates its performance on the testing data.

For manuscripts utilizing custom algorithms or software that are central to the research but not yet described in published literature, software must be made available to editors and reviewers. We strongly encourage code deposition in a community repository (e.g. GitHub). See the Nature Research guidelines for submitting code & software for further information.

Data

Policy information about availability of data

All manuscripts must include a data availability statement. This statement should provide the following information, where applicable:

- Accession codes, unique identifiers, or web links for publicly available datasets
- A list of figures that have associated raw data
- A description of any restrictions on data availability

All data generated or analysed during this study are included in this published article and its supplementary information files. The source data necessary to reproduce Figs. 1, 2, 3, 4, 5; Tables 1 and 2; and Supplementary Figs. C1, C2, C3,C4, C5, C6 and Tables C1, C2, C3, C4, C5 and C6 are provided as a Source Data file.

April 2020

Field-specific reporting

Please select the one below that is the best fit for your research. If you are not sure, read the appropriate sections before making your selection.

☐ Life sciences ☒ Behavioural & social sciences ☐ Ecological, evolutionary & environmental sciences

For a reference copy of the document with all sections, see nature.com/documents/nr-reporting-summary-flat.pdf

Behavioural & social sciences study design

All studies must disclose on these points even when the disclosure is negative.

Study description	We did an online survey experiment which corresponds to quantitative research.
Research sample	The online survey experiment was conducted among the general public of Spain (N = 2004). The data was collected using quotas on age, gender and geographical regions to achieve a nationally representative sample on these socio-demographic dimensions. Thus, our sample has 51.1% of females, the mean age was 45.15 and 89.92% of the sample have medium professional or higher studies (see Table C1 of the Appendix for further information regarding demographic information of the sample). We use quotas on age, gender and geographical distribution to ensure similarity among treatment and control groups.
Sampling strategy	The company that implemented the survey chose respondents from a panel randomly applying quotas on age, gender and geographical regions to achieve a nationally representative sample on these socio-demographic dimensions. A sample size of >1000 respondents is widely considered as sufficient to achieve a representative sample of a country with the population of Spain.
Data collection	The data was collected through an online questionnaire by a company, Netquest, which was the one in charge of the recruitment, compensation and provision of anonymised final data file to the researchers. The company has the certification ISO 26362 which regulates certain aspects such as respondents explicit informed consent and the privacy policy of the company. Data was recorded in an Excel file.
Timing	The data was collected by a professional survey company during August 2019
Data exclusions	The survey was sent to 3415 Spanish citizens. A total amount of 2534 people accessed the survey, among whom 530 persons were filtered out of the sample, for different reasons, such as answering the control questions incorrectly (11), leaving the survey without completing it (355), or because the quota to which they belonged was already completed (123). This resulted in a final sample size of 2004 and a response rate of 58,68%.
Non-participation	355 respondents left the survey without completing it. There were no participants declining participation although there were 881 citizens that received the invitation to participate in the survey but did not accessed it.
Randomization	We divided our sample into two sub-samples. We use quotas on age, gender and geographical distribution to ensure similarity among treatment and control groups. In addition, we tested that these samples do not differ on other covariates, such as climate concern and education (see Table C.2 in Appendix C).

Reporting for specific materials, systems and methods

We require information from authors about some types of materials, experimental systems and methods used in many studies. Here, indicate whether each material, system or method listed is relevant to your study. If you are not sure if a list item applies to your research, read the appropriate section before selecting a response.

Materials & experimental systems

n/a	Involved in the study	
☒	☐	Antibodies
☒	☐	Eukaryotic cell lines
☒	☐	Palaeontology and archaeology
☒	☐	Animals and other organisms
☐	☒	Human research participants
☒	☐	Clinical data
☒	☐	Dual use research of concern

Methods

n/a	Involved in the study	
☒	☐	ChIP-seq
☒	☐	Flow cytometry
☒	☐	MRI-based neuroimaging

Human research participants

Policy information about studies involving human research participants

Population characteristics	Sampling was done by using quotas on age, gender and geographical distribution, making the survey sample representative of the general population of Spain on these characteristics. 51.1% were women and the mean age was 45.15 years old. The

	results show that the sample is also representative for the other covariates, except for the variable education. The mean household size is of 2.96 individuals, the mean monthly household income is between €2200 and 2700 and our sample is situated slightly to the left concerning political orientation. With regards to education, 89.92% of the sample have medium professional or higher studies, which is higher than the average of the Spanish population (60.2%).
Recruitment	Respondents were part of a panel from the company that implemented the questionnaire. The company contacted participants from the panel randomly applying quotas on age, gender and geographical regions to achieve a nationally representative sample on these socio-demographic dimensions. The survey was sent to 3415 Spanish citizens. A total amount of 2534 people accessed the survey, among whom 530 persons were filtered out of the sample, for different reasons, such as answering the control questions incorrectly (11), leaving the survey without completing it (355), or because the quota to which they belonged was already completed (123). This resulted in a final sample size of 2004 and a response rate of 58,68%. As is common in quota sampling surveys, self-selection bias may result; in this case due to, e.g., overrepresentation of people with a relatively strong interest in climate issues. The company has the certification ISO 26362 which recommends fair and balanced rewards for panelists. This means that the incentive system must be public and documented and should be as neutral as possible to avoid biases in the results of the project. As a panelist, respondents will obtain conches that can be exchanged for gifts in the company online store whenever respondents participate in surveys.
Ethics oversight	The survey was approved by the Autonomous University of Barcelona's Committee on Ethics in Animal and Human Experiments.

Note that full information on the approval of the study protocol must also be provided in the manuscript.
